# Cyclooxygenase-2 (COX-2) Inhibition Constrains Indoleamine 2,3-Dioxygenase 1 (IDO1) Activity in Acute Myeloid Leukaemia Cells

**DOI:** 10.3390/molecules180910132

**Published:** 2013-08-22

**Authors:** Maria Grazia Iachininoto, Eugenia Rosa Nuzzolo, Giuseppina Bonanno, Andrea Mariotti, Annabella Procoli, Franco Locatelli, Raimondo De Cristofaro, Sergio Rutella

**Affiliations:** 1 Department of Haematology, Catholic University Medical School, Largo A. Gemelli 8, 00168 Rome, Italy; E-Mails: mg.iachininoto@rm.unicatt.it (M.G.I.); eugenia.nuzzolo@gmail.com (E.R.N.); 2 Department of Gynaecology and Obstetrics, Catholic University Medical School, Largo A. Gemelli 8, 00168 Rome, Italy; E-Mails: giuseppina.bonanno@rm.unicatt.it (G.B.); andrea.mariotti@rm.unicatt.it (A.M.); a.procoli@libero.it (A.P.); 3 Department of Pediatric Haematology/Oncology and Transfusion Medicine, IRCCS Bambino Gesù Children’s Hospital, Piazza Sant’Onofrio 4, 00165 Rome, Italy; E-Mail: franco.locatelli@opbg.net (F.L.); 4 Department of Pediatrics, University of Pavia, Strada Nuova 65, 27100 Pavia, Italy; 5 Department of Medicine and Geriatrics, Catholic University Medical School, Largo A. Gemelli 8, 00168 Rome, Italy; E-Mail: rdecristofaro@rm.unicatt.it

**Keywords:** indoleamine 2-3-dioxygenase, immune tolerance, acute leukaemia, regulatory T cells, immunotherapy, interferon-γ

## Abstract

Indoleamine 2,3-dioxygenase 1 (IDO1) metabolizes L-tryptophan to kynurenines (KYN), inducing T-cell suppression either directly or by altering antigen-presenting-cell function. Cyclooxygenase (COX)-2, the rate-limiting enzyme in the synthesis of prostaglandins, is over-expressed by several tumours. We aimed at determining whether COX-2 inhibitors down-regulate the IFN-γ-induced expression of IDO1 in acute myeloid leukaemia (AML) cells. IFN-γ at 100 ng/mL up-regulated COX-2 and IDO1 in HL-60 AML cells, both at mRNA and protein level. The increased COX-2 and IDO1 expression correlated with heightened production of prostaglandin (PG)E_2_ and kynurenines, respectively. Nimesulide, a preferential COX-2 inhibitor, down-regulated IDO1 mRNA/protein and attenuated kynurenine synthesis, suggesting that overall IDO inhibition resulted both from reduced *IDO1* gene transcription and from inhibited IDO1 catalytic activity. From a functional standpoint, IFN-γ-challenged HL-60 cells promoted the *in vitro* conversion of allogeneic CD4^+^CD25^−^ T cells into *bona fide* CD4^+^CD25^+^FoxP3^+^ regulatory T cells, an effect that was significantly reduced by treatment of IFN-γ-activated HL-60 cells with nimesulide. Overall, these data point to COX-2 inhibition as a potential strategy to be pursued with the aim at circumventing leukaemia-induced, IDO-mediated immune dysfunction.

## 1. Introduction

Indoleamine 2,3-dioxygenase 1 (IDO1) has become a recognized mediator of immune tolerance in cancer-bearing hosts, promoting local metabolic changes that affect cellular and systemic responses to inflammatory and immunological signals [1]. Tryptophan metabolism mediated by IDO1 generates biologically active kynurenine pathway metabolites, leading to differentiation and/or activation of FoxP3-expressing regulatory T cells (Treg), to suppression of anti-tumour T-cell responses and to reduced dendritic cell (DC) immunogenicity [2,3]. The rapid consumption of tryptophan from the local microenvironment triggers the activation of molecular stress-response pathways, such as those involving the GCN2 kinase [4], leading to cell cycle arrest and anergy in CD8^+^ T cells, and to regulatory T-cell (Treg) differentiation and inhibition of Th17 cytokine secretion in CD4^+^ T cells [1]. Constitutive expression of IDO is detected primarily at mucosal sites, but the IDO1 pathway is induced in many tissues during inflammation, as *IDO1* gene expression is tightly regulated by interferon (IFN)-γ [5]. Early reports suggested that all 3 types of IFN (α, β and γ) may induce IDO [6]. However, normal and transformed cell lines were shown to express IDO in response to IFN-γ, but not to IFN-α or IFN-β [5]. When tested *in vitro*, lung fibroblasts and bladder carcinoma cell lines did not express IDO in response to either IFN-α or IFN-β [7]. Similarly, IDO mRNA in human fibroblasts is induced by IFN-α but transiently and with lower potency when compared with IFN-γ [8].

IDO1 is also expressed by a variety of tumours [9] and by antigen-presenting cells (APC), including macrophages, human monocyte-derived DC, and individual subsets of murine DC [10,11]. IDO1 activity in tumour cells may reflect either constitutive IDO1 protein expression [12] or IDO1 induction as a consequence of microenvironmental interactions [9]. In this respect, gastric carcinoma, colon carcinoma and renal cell carcinoma cell lines do not express IDO1 constitutively but rather they up-regulate the enzyme following IFN-γ stimulation [13]. Importantly, IDO1 confers an unfavourable prognosis to a variety of solid tumours and to acute myeloid leukaemia (AML) [14,15]. In patients with AML, a higher serum kynurenine/tryptophan ratio correlates with shorter survival [16]. Also, high *IDO* mRNA levels in blast cells of adult patients with AML predict a poor clinical outcome [14].

Prostaglandin (PG) G/H synthases, referred to as COX, exist in two isoforms, COX-1 and COX-2, and convert arachidonic acid into a biologically inactive unstable intermediate, the PGH_2_, which is further converted by cell-specific synthases into biologically active end-products, collectively known as prostanoids [17]. Like IDO1, COX-2 can be induced by IFN-γ in several cell types [18]. Importantly, COX-2 is constitutively over-expressed by epithelial malignancies, including human non-small cell lung cancer, where it confers a malignant and metastatic phenotype [19]. This phenomenon is largely due to overproduction of PGE_2_, a prostanoid that enhances cell proliferation, invasion, metastasis and angiogenesis, and inhibits apoptosis [20,21,22]. Although the molecular mechanisms implicated in COX-2 up-regulation in leukaemia cells remain elusive, there is evidence that blast cells from at least some subsets of patients with AML express functional COX-2 in response to an array of stimuli [23]. The ability of COX-2 to promote escape from immunosurveillance is exemplified by the observation that inhibition of COX-2/PGE_2_ in mice with lung cancer reduces Treg-cell frequencies and increases the number of CXCR3^+^, anti-tumour effector T cells [24]. The interplay between COX-2 and IDO1 is further underpinned by studies in animal models of cancer, where pharmacological blockade of COX-2 translated into down-regulation of IDO1 expression at the tumour site, leading to decreased serum kynurenines [25,26]. It is presently unknown whether IFN-γ-induced COX-2 may also regulate IDO1 expression in human leukaemia cells. 

## 2. Results and Discussion

### 2.1. Induction of Functionally Active COX-2 and IDO1 by IFN-γ in HL-60 Cells

**Figure 1:**
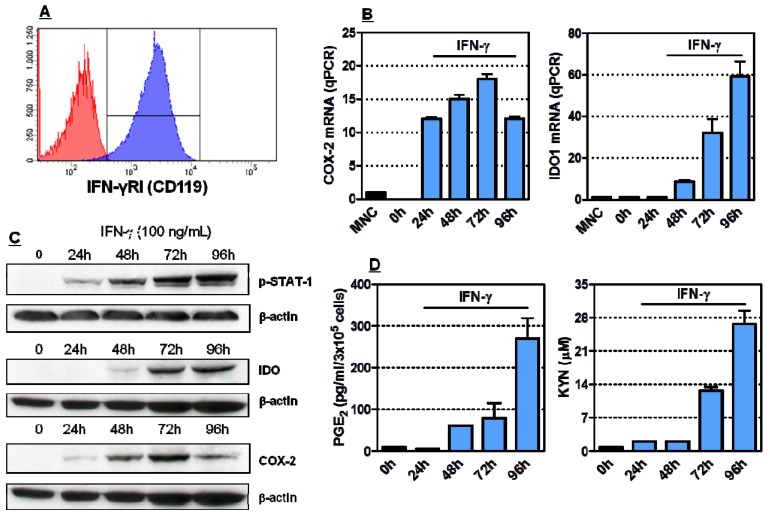
Induction of COX-2 and IDO1 in HL-60 leukaemia cells. Panel A: The expression of IFN-γ receptor I (CD119) was investigated by flow cytometry. One representative experiment out of 4 with similar results is shown. Panel B: Quantitative RT-PCR was conducted to measure COX-2 and IDO1 mRNA levels in IFN-γ challenged HL-60 leukaemia cells. Graphs summarize 5 independent experiments. Data are expressed in terms of mean ± SEM. Panel C: HL-60 cells were activated with 100 ng/mL IFN-γ for up to 96 h, followed by Western blot runs to detect phosphorylated STAT1, COX-2 and IDO1 proteins. Panel D: Measurement of PGE_2_ and kynurenines in supernatants of HL-60 leukaemia cells stimulated with 100 ng/mL IFN-γ. Bars reflect the mean and SEM recorded in 3 independent experiments.

IFN-γ signalling occurs through the JAK-STAT pathway, leading to the phosphorylation of STAT-1α and to its translocation to the nucleus, where it initiates transcription [27]. We first investigated whether HL-60 cells express receptors for IFN-γ. Cells were analyzed by flow cytometry after labelling with anti-CD119 monoclonal antibodies (mAb) that react against the IFN-γ receptor α chain, required for ligand binding and for signalling [28]. As shown in Figure 1A, HL-60 cells expressed readily detectable levels of CD119 antigen on the cell surface. HL-60 cells were then incubated with IFN-γ, a prototypical inducer of IDO in normal and tumour cell types [6], for up to 96 h. For polymerase chain reaction (PCR) studies, RNA was isolated, converted to cDNA and amplified for COX-2 and IDO1. Equivalence of RNA loading was verified by the consistency of mRNA housekeeping signals (data not shown). As illustrated in Figure 1B, COX-2 transcripts were detected within 24 h after incubation with IFN-γ, and steadily increased at 72 h and 96 h, in accordance with previous reports suggesting the ability of HL-60 cells to express COX-2 after culture with doxorubicin or with lipopolysaccharide [23,29]. Conversely, IDO1 mRNA was detected in HL-60 AML cells 48 h after IFN-γ provision, and remained stably expressed for up to 3 days. Changes in COX-2 expression after leukaemia cell stimulation with IFN-γ would be suggestive of the involvement of the COX-2 system in IDO1 induction. To analyze the effects of IFN-γ on COX-2 and IDO1 expression and regulation, we performed western blot analyses using COX-2-specific and IDO1-specific antibodies. Not unexpectedly, blotting with an antibody specific to the transcriptionally active, phosphorylated form of STAT-1 showed that phospho-STAT-1 was rapidly induced by IFN-γ in leukaemia cells (Figure 1C). COX-2 protein was detected after 24 h of IFN-γ treatment, peaked at 72 h and declined thereafter (Figure 1C). IFN-γ-treated HL-60 cells also expressed IDO1 protein, with maximum levels being detectable after 72 h from the addition of IFN-γ to the culture medium, as shown in Figure 1C. The release of PGE_2_, a prostanoid that is dependent on COX-2 activity, was measured by ELISA, whereas kynurenine and tryptophan levels were quantified with RP-HPLC. Importantly, supernatants of IFN-γ-treated HL-60 cells were significantly enriched both in PGE_2_ and in kynurenine compared with supernatants collected from cytokine-untreated cells and with culture medium alone (Figure 1D). In accordance with this finding, tryptophan was depleted in supernatants of IFN-γ-stimulated HL-60 cells (data not shown), pointing to the IDO-mediated activation of tryptophan metabolism. Collectively, these experiments indicated that COX-2 mRNA and protein are up-regulated by IFN-γ in leukaemia cells and that IFN-γ also induces functional IDO1 in HL-60 cells.

### 2.2. COX-2 Inhibition Restrains IDO1 Activity in AML Cells

**Figure 2:**
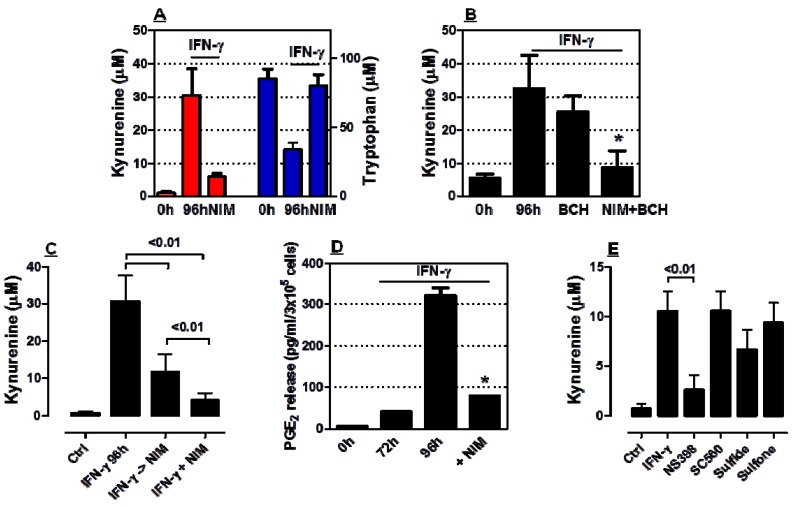
Modulation of IDO1 activity by COX-2 inhibitors. Leukaemia cells were stimulated with 100 ng/ml IFN-γ for 72 h, followed by the exposure to 100 µM nimesulide (NIM) for 24 h. Panel A: Kynurenine and tryptophan in culture supernatants (mean and SEM from 7 independent experiments). Panel B: Kynurenine levels after the provision of either nimesulide (NIM) or BCH, an inhibitor of amino acid transport, to leukaemia cells (mean and SEM from 3 independent experiments). * Denotes a *p* value < 0.01 compared with cultures maintained without nimesulide. Panel C: Kynurenine levels after simultaneous or sequential provision of IFN-γ and NIM to leukaemia cells. Panel D: PGE_2_ levels after provision of NIM to IFN-γ-challenged leukaemia cells. Bars reflect the mean and SEM recorded in 3 independent experiments. * Denotes a *p* value < 0.01 compared with cultures maintained without nimesulide. Panel E: Kynurenine release after the provision of selective COX-2 (NS398) or COX-1 inhibitors (SC560, sulindac sulfide) to leukaemia cells. Bars reflect the mean and SEM recorded in 3 independent experiments.

We next cultured HL-60 AML cells with nimesulide, a preferential COX-2 inhibitor, after their challenge with IFN-γ for 72 h. As shown in Figure 2A, nimesulide almost completely abrogated kynurenine release by IFN-γ-activated AML cells. In line with these results, tryptophan was significantly depleted from the supernatant of AML cell cultures containing IFN-γ. BCH is a synthetic aminoacid that inhibits tryptophan influx through the system L transporter, which mediates a limiting step for IDO1-activated L-tryptophan degradation in placental tissues [30]. When added to HL-60 cells at 2 mM, a concentration that, by itself, has not been reported to affect significantly IDO enzymatic activity [27], BCH exerted but minimal effects on kynurenine release by IFN-γ-activated AML cells (Figure 2B). 

Importantly, nimesulide also inhibited IDO-mediated tryptophan breakdown in cultures containing BCH, suggesting that nimesulide affected IDO1 catalytic activity rather than limiting substrate availability. We also measured kynurenine levels in cultures performed with AML cells that were either concurrently treated with IFN-γ and nimesulide for 96 h or sequentially exposed to IFN-γ for 72 h followed by further 24 h of culture with nimesulide. Figure 2C summarizes the results of these experiments by showing that simultaneous provision of nimesulide and IFN-γ to AML cells translated into an even more remarkable inhibition of IDO1 enzymatic activity compared with cultures that were first treated with IFN-γ to induce IDO1 and then incubated with nimesulide to inhibit COX-2. Nimesulide also down-regulated PGE_2_ release in supernatants of AML cells compared with IFN-γ-stimulated AML cells that were maintained in the absence of COX-2 inhibitors (Figure 2D). 

In a further set of experiments, we treated IFN-γ-stimulated AML cells with NS398, a selective and potent COX-2 inhibitor. Control cultures contained either SC560 or sulindac sulfide, which preferentially inhibit COX-1 activity. Sulindac sulfone, another metabolite of sulindac sulfoxide that has anticancer properties but lacks COX inhibitory activity, was used in selected experiments. As shown in Figure 2E, the provision of NS398 to IFN-γ-stimulated AML cells translated into a significant reduction of kynurenine levels in culture supernatants. In sharp contrast, COX-1 inhibitors only marginally affected kynurenine levels. As expected based on lack of COX inhibitory activity, sulindac sulfone had no measurable impact on the IFN-γ-induced release of kynurenine in culture supernatants.

### 2.3. COX-2 Inhibition Regulates IDO1 Expression in AML Cells Both at Transcriptional and Post-Transcriptional Level

**Figure 3:**
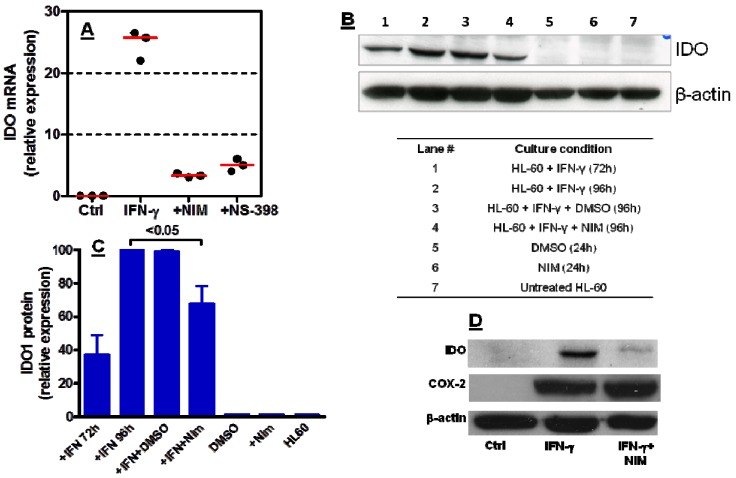
Modulation of IDO1 mRNA and protein by COX-2 inhibitors. Leukaemia cells were stimulated with 100 ng/mL IFN-γ for 72 h, followed by the exposure to COX-2 inhibitors for 24 h. Panel A: IDO mRNA expression by leukaemia HL-60 cells as assessed with quantitative RT-PCR. Panel B: IDO protein expression by leukaemia HL-60 cells in a representative experiment out of five with similar results. Panel C: Densitometry of western blot runs; bars depict the mean and SEM from five independent experiments. Culture conditions and lane numbers for the experiments shown in panels B and C are detailed at the bottom of panel B. Panel D: COX-2 and IDO protein expression by leukaemia HL-60 cells that were stimulated with IFN-γand then treated with nimesulide for 24 h. A representative experiment out of 3 with similar results is shown.

It has been shown that treatment of tumour-bearing PyV MT mice with celecoxib, a selective COX-2 inhibitor, reduces IDO protein levels and augments the efficacy of DC-based breast cancer vaccines [26]. In a further set of experiments, we aimed at addressing whether COX-2 may regulate IDO1 expression at either the mRNA or protein level. We used quantitative RT-PCR to measure IDO1 mRNA in IFN-γ-challenged leukaemia cells. As Figure 3A–C show, nimesulide significantly down-regulated IDO mRNA and protein in HL-60 AML cells. To assess whether IDO1 regulation by nimesulide occurred as a result of reduced COX-2 expression, HL-60 cells were initially activated with IFN-γ in the presence or absence of nimesulide, and then subjected to Western blot runs with COX-2- specific antibodies. Interestingly, COX-2 protein levels were unchanged in nimesulide-treated, IFN-γ-activated HL-60 cells compared with cultures maintained with IFN-γ alone, suggesting lack of modulation of COX-2 protein levels by the COX-2 inhibitor (Figure 3D). Recently, reduced intra-tumour expression of IDO and lowered PGE_2_ serum levels have been detected in mice with syngeneic lung cancer that were given COX-2 inhibitors [31]. Along the same line of research, it has been elegantly shown that mice with pancreatic adenocarcinoma treated with anti-tumour vaccines and celecoxib experience reduced COX-2 and IDO1 function, as manifested by lowered serum levels of PGE_2_ and kynurenine, respectively [25]. Taken together with the above mentioned *in vivo* studies, our findings point to the relevance of COX-2-mediated regulation of IDO1 expression in tumour cells at both transcriptional and post-transcriptional level.

### 2.4. COX-2 Inhibition Restrains the *in Vitro* Generation of Bona fide Treg Cells by IFN-γ-Stimulated HL-60 Cells

**Figure 4:**
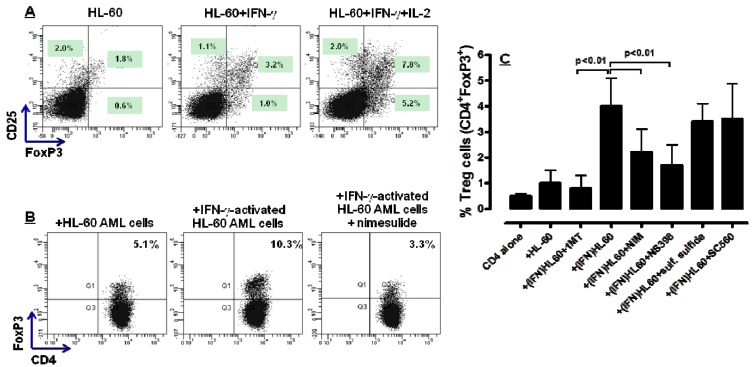
Effect of COX-2 inhibitors on leukaemia cell-induced differentiation of *bona fide* Treg cells. HL-60 cells were stimulated with 100 ng/mL IFN-γ for 72 h, followed by co-culture with allogeneic naive CD4^+^CD25^−^ T cells for 5 days in a mixed tumour-lymphocyte culture (MTLC). Panel A: Expression of CD25 and intracellular FoxP3 after co-culture with HL-60 AML cells in a representative experiment. IL-2 was provided to the cultures as a Treg-specific growth factor. Panel B: Expression of intracellular FoxP3 by CD4^+^ T cells after co-culture with HL-60 AML cells either in the absence or presence of 100 mM nimesulide (one representative experiment is shown). Panel C: Cumulative results of co-culture experiments (n = 5), depicting the inhibition of Treg differentiation by AML cells that were also cultured with selective COX-2 (NS398) or COX-1 inhibitors (SC560, sulindac sulfide). In selected experiments, 1MT, a chemical inhibitor of IDO, was added at 200 μM (final concentration) during the MTLC. Bars depict mean and SEM.

FoxP3 is a transcription factor and master regulatory of Treg differentiation and function [32]. Moreover, the frequency of FoxP3^+^ Treg cells is increased in most tumours [33] and has been correlated with COX-2 activity in solid cancers [34]. We then asked whether COX-2 inhibition exerted any appreciable effect on the *in vitro* differentiation of Treg cells from naive T cells by IDO-expressing HL-60 AML cells. To this end, HL-60 cells were stimulated with exogenous IFN-γ for 72 h, followed by 24 h of culture with either nimesulide or PBS as a control culture condition. HL-60 were then plated with allogeneic CD4^+^CD25^−^ T cells for 5 days in a mixed tumour- lymphocyte culture (MTLC). As shown in Figure 4A, IFN-γ-challenged HL-60 cells promoted the *in vitro* conversion of naive CD4^+^ T cells into *bona fide* Treg cells, an effect that was potentiated by the provision of IL-2, a Treg growth factor [35]. Interestingly, the frequency of FoxP3-expressing T cells was significantly lower in MTLC performed with HL-60 that were treated with nimesulide during IFN-γ challenge. A representative experiment out of 3 with similar results is shown in Figure 4B. When providing the MTLC with HL-60 leukaemia cells that were previously treated with NS398, Treg differentiation was also inhibited, at variance with co-cultures containing AML cells that were maintained with preferential COX-1 inhibitors, such as SC560, or with sulfone sulfide. The cumulative data from this set of experiments is summarized in Figure 4C. It should be emphasized that the inhibition of Treg differentiation by HL-60 cells pre-treated with nimesulide was not maximal, at variance with that attained with 1MT, a chemical IDO inhibitor currently in phase I clinical trials for patients with advanced cancer [36]. This interpretation of the data implies that both IDO1 and COX-2 activities may be required to promote Treg development by HL-60 leukaemia cells. Collectively, cellular assays suggested that COX-2 inhibition interferes, albeit incompletely, with the *in vitro* generation of Treg cells by leukaemia cells. The favourable effects of constrained Treg expansion on the anti-leukaemia immune response have been unravelled in immune-competent mice bearing A20 leukaemia, where IDO1 inhibitors were shown to promote disease control [37].

## 3. Experimental

### 3.1. Cells and Reagents

HL-60 AML cells were obtained from American Type Culture Collection (No CCL-240, ATCC, Manassas, VA, USA). IFN-γ was obtained from R&D Systems, Oxon, Cambridge, UK. The following antibodies were used: mouse monoclonal antibodies (mAb) against anti-IDO (clone 10.1; Millipore, Billerica, MA, USA), mouse mAb against COX-2, rabbit polyclonal anti-p-STAT-1(Tyr701) antibodies and mouse mAb against β-actin (Santa Cruz Biotechnology, Heidelberg, Germany). 2-Amino-2-norbornanecarboxylic acid (BCH), an amino acid transport inhibitor (2 mM final concentration) and nimesulide (100 µM final concentration) were purchased from Sigma Chemical Co. (St. Louis, MO, USA). The following compounds were obtained from Merck Chemicals Ltd. (Nottingham, UK): SC-560 [5-(4-Chlorophenyl)-1-(4-methoxyphenyl)-3-trifluoromethylpyrazole], sulindac sulfide (a metabolite of the nonsteroidal anti-inflammatory drug sulindac sulfoxide and a cell-permeable selective COX-1 inhibitor), and NS-398 [N-(2-Cyclohexyloxy-4-nitrophenyl)-methanesulfonamide, a cell-permeable selective COX-2 inhibitor]. Sulindac sulfone, a cell-permeable sulfone metabolite of sulindac sulfoxide which lacks COX inhibitory activity (Merck Chemicals Ltd.), was used in control experiments. Each COX inhibitor drug was used at a concentration 10 times higher than the IC_50_ value reported by the manufacturer.

### 3.2. Induction of IDO1 in Leukaemia HL-60 Cells

HL-60 AML cells were maintained in culture with Iscove’s Modified Dulbecco’s Medium (IMDM; Life Technologies BRL, Rockville, MD, USA) supplemented with 20% foetal bovine serum (FBS), penicillin-streptomycin (EuroClone, Milan, Italy) and 2 mM L-glutamine (Life Technologies). Cells were grown in 25 cm^2^ culture flasks (Corning, Corning, NY, USA) at 37 °C in a 5% CO_2_ humidified atmosphere, and cell density was maintained at 1 × 10^5^–10^6^ viable cells by replacing medium every 2–3 days. HL-60 cells were exposed to exogenous IFN-γ for up to 96 h. After culturing, cells were recovered, counted and subjected to quantitative real-time (RT)-PCR for the detection of COX-2 and IDO1 mRNA or to western blotting experiments, as detailed below. For COX-2 inhibition, HL-60 cells (1 × 10^5^/mL) were stimulated for 72 h with 100 ng/mL recombinant human IFN-γ and then treated for 24 h with either of the following reagents: 100 µM nimesulide, 50 µM NS-398, 50 µM sulindac sulfide, 50 µM sulindac sulfone or DMSO (solvent). Cell viability after treatment with inhibitors was estimated with trypan blue exclusion dye (EuroClone).

### 3.3. T-Cell Isolation and Primary MLR

Mononuclear cells from healthy consented subjects were isolated by Ficoll-Hypaque density gradient, as previously published [38]. CD25^+^ cells were purified by positive selection using directly conjugated anti-CD25 magnetic microbeads (4 μL per 10^7^ cells; Miltenyi Biotec, Bergisch Gladbach, Germany). After the double column procedure, CD4^+^CD25^+^ cells were routinely more than 94% pure by FACS analysis (data not shown). The remaining non-CD25^+^ fraction was used for the isolation of CD4^+^CD25^−^ cells by positive selection with anti-CD4 mAb-coated microbeads (Miltenyi Biotec). CD4^+^CD25^−^ T cells were cultivated with HL-60 AML cells in mixed lymphocyte-tumour cultures (MLTC).

### 3.4. Immunological Markers and Flow Cytometry

Cells were incubated for 20 min at 4 °C with fluorochrome-conjugated anti-CD4, anti-CD25 and anti-CD119 mAb (all from BD Biosciences, Mountain View, CA, USA) or with fluorochrome-conjugated, isotype-matched irrelevant mAb to establish background fluorescence. The frequency of *bona fide* Treg cells was determined with the Human Regulatory T-Cell Staining Kit (eBioscience, San Diego, CA, USA). Briefly, cells were initially labelled with anti-CD4 and anti-CD25 mAb, followed by sequential fixation and permeabilization, and by staining with Alexa Fluor^®^ 488-conjugated anti-FoxP3 mAb (PCH101 clone). All samples were run through a FACS Canto^®^ II flow cytometer (BD Biosciences) with standard equipment, as already detailed [11].

### 3.5. Measurement of PGE_2_ by ELISA

PGE_2_ levels in culture supernatants were measured with a commercially available ELISA kit (R&D Systems). The minimum detectable dose of PGE_2_, as evaluated by the manufacturer, ranges between 18.2 and 36.8 pg/mL.

### 3.6. Western Blotting

After treatment with IFN-γ, 1 × 10^6^ HL-60 cells were re-suspended with PBS and centrifuged at 1,200 rpm for 10 min. Cell pellets were lysed with RIPA buffer and protease inhibitors (Sigma Chemicals). Cell lysates were incubated for 5 minutes on ice, and then clarified by centrifugation at 13,000 rpm for 20 min. Once obtained, cell extracts were boiled for 5 min at 95 °C and total lysate (40 µg) was analyzed by 10% sodium dodecyl sulphate-polyacrylamide gel electrophoresis (SDS-PAGE). Samples were then transferred onto a nitrocellulose membrane (Amersham Hybond; GE Healthcare, Milan, Italy). After blocking non-specific binding, blots were probed overnight at 4 °C with either primary mouse mAb directed against IDO (2 µg/mL) or COX-2 (1:100 dilution), or rabbit polyclonal anti-p-STAT-1(Tyr701) (1:200 dilution) or mouse mAb directed against β-actin (1:2,000 dilution). The membrane was then incubated with horseradish peroxidase-conjugated rabbit or mouse secondary antibodies (Sigma) as appropriate, and the chemiluminescence reaction was detected with Amersham ECL-western blotting detection reagents (GE Healthcare). Densitometry was performed by quantifying the bands with a freely available image analysis tool written at the National Institutes of Health (Scion Image for Windows 4.03).

### 3.7. Quantitative RT-PCR

Total RNA was extracted from HL-60 cells using the RNeasy mini kit (Qiagen, Milan, Italy), according to the manufacturer’s instructions. Complementary DNA (cDNA) was prepared starting from 1 µg of total RNA using Moloney Murine Leukemia Virus (MMLV) reverse transcriptase and random hexamer primers (Promega, Milan, Italy). Gene expression levels were quantified by a SYBR Green-based real-time method. Reactions were carried out in triplicate in a final volume of 25 µL containing 2 µL of cDNA, iQ SYBR Green Supermix (2×) (Bio-Rad Laboratories, Milan, Italy) and 250 nM of each primer. Amplifications were carried out using specific primers for the *IDO1* gene (forward primer 5'-3' GGTCATGGAGATGTCCGTAA; reverse primer 5'-3' ACCAATAGAGAGAC CAGGAAGAA) or the *COX2* gene (forward primer 5'-3' CCTGCCCTTCTGGTAGAAA; reverse primer 5'-3' GGACAGCCCTTCACGTTATT). GAPDH served as a housekeeping gene (forward primer 5'-3': TCCCTGAGCTGAACGGGAAG; reverse primer 5'-3': GGAGGAGTGGGTGTCGTCG CTGT). Thermal cycling was performed with the iCycler iQ system (Bio-Rad Laboratories) using 60 °C as annealing temperature. All quantifications were normalized to the reference gene and expressed using the ΔCt method.

### 3.8. IDO1 Activity

Tryptophan and kynurenine levels were measured with reverse-phase (RP)-HPLC. Briefly, sample aliquots were deproteinized with 0.3 M HClO_4_. Supernatants were spiked with 50 µM 3-L-nitrotyrosine and analyzed using a ReproSil-Pur C18-AQ RP-HPLC column (Dr. Maisch GmbH, Ammerbuch-Entringen, Germany), using a double-pump HPLC apparatus from Jasco (Tokyo, Japan) equipped with spectrophotometric and fluorescence detectors. The chromatographic peaks were detected by recording UV absorbance at 360 nm and emission fluorescence at 366 nm, after excitation at 286 nm. The elution solvent was as follows: 2.7% CH_3_CN in 15 mM acetate buffer, pH 4.0 (both HPLC-grade; Fluka, Milan, Italy). The Borwin 1.5 and MS Excel software packages were used for instrument set-up and peak quantification. The concentration of components was calculated according to peak heights and was compared both with 3-nitro-L-tyrosine as internal standard and with reference curves constructed with L-tryptophan and kynurenine.

### 3.9. Statistical Analysis

The approximation of data distribution to normality was preliminarily tested with statistics for kurtosis and symmetry. Results were presented as mean and SD. All comparisons were performed with the Student’s *t* test for paired or unpaired determinations or with the analysis of variance (ANOVA), as appropriate. The criterion for statistical significance was defined as *p* ≤ 0.05.

## 4. Conclusions

The interactions between AML cells and the immune system contribute to the establishment of immune tolerance against malignant cells [39]. The present study suggests that the regulation of IDO1 expression by inhibition of the COX-2/PGE_2_ pathway may constrain the leukaemia-induced immune suppression. It has been demonstrated that blasts from some patients with AML have the ability to up-regulate COX-2 expression in response to pro-inflammatory stimuli [23]. Conceivably, chronic inflammation associated with cancer may create immune suppression through the up-regulation of IDO1, but locally in the bone marrow microenvironment and systemically [40,41]. Our findings thus portend favourable implications for immunotherapy trials aimed at reinstituting anti-leukaemia immune responses through the inhibition of the COX-2/IDO1 axis.
